# Conventional aortic valve replacement can be safely done by very early stage trainee

**DOI:** 10.3389/fsurg.2025.1603896

**Published:** 2025-07-10

**Authors:** Kentaro Shirakura, Nobuhiro Mochizuki, Ryohei Ushioda, Shingo Kunioka, Masahiro Tsutsui, Hiroyuki Kamiya

**Affiliations:** Department of Cardiac Surgery, Asahikawa Medical University, Asahikawa, Japan

**Keywords:** AVR, SAVR, trainee, cardiac surgeon, aortic valve

## Abstract

**Objectives:**

We have continuously performed conventional aortic valve replacement (AVR) with median sternotomy as the primary approach because we believe that it is the safest approach, and even very young trainees have performed surgical AVR (SAVR) under proper supervision. Here we reviewed our results of AVR to clarify whether our aggressive training program would be justified.

**Methods:**

This retrospective study evaluates the outcomes of trainee surgeons performing SAVR under supervision at a single institution. We analyzed 145 patients who underwent isolated SAVR between January 2015 and April 2024. Patients were divided into two groups: those operated on by staff surgeons with more than 7 years of postgraduate experience in the Japanese residency program (*n* = 91), and those operated on by resident surgeons with 2–6 years of postgraduate experience in the Japanese residency program (*n* = 54). Outcomes compared preoperative characteristics, intraoperative metrics, postoperative complications, and survival rates.

**Results:**

Results showed no significant difference in operative time, and aortic cross-clamp time between the groups. Furthermore, early postoperative mortality and mid-term survival rates were comparable. Although staff surgeons had higher Japan SCORE, residents demonstrated similar clinical outcomes.

**Conclusions:**

SAVR can be safely performed by very early-stage trainees under proper case selection and supervision.

## Introduction

Surgical units are increasingly facing the challenge of balancing the need to train young surgeon with the duty to serve the highest standard of patient care. In recent years, the number of patients presenting with more comorbidities has been increasing and requiring more complex procedures (1, 2). Thus, the surgical opportunity and responsibilities of trainees may be limited because of the perceived increased risk of possible complications (3–5). Aortic valve replacement (AVR) via median sternotomy is a highly reproducible procedure with stable outcomes, therefore it has been considered to be suitable for less experienced trainee, however, increasing number of transcatheter aortic valve implantation (TAVI) and minimally invasive aortic valve replacement (MIAVR) has made the situation regarding surgical education more difficult (6–9). At our institute, we have continuously performed conventional AVR with median sternotomy as the primary approach for patients to be indicated for surgical AVR (SAVR) because we believe that it is the safest approach, and even very young trainees have performed AVR under proper supervision. Here we reviewed our results of AVR to clarify whether our aggressive training program would be justified.

## Materials and methods

**Figure 1:**
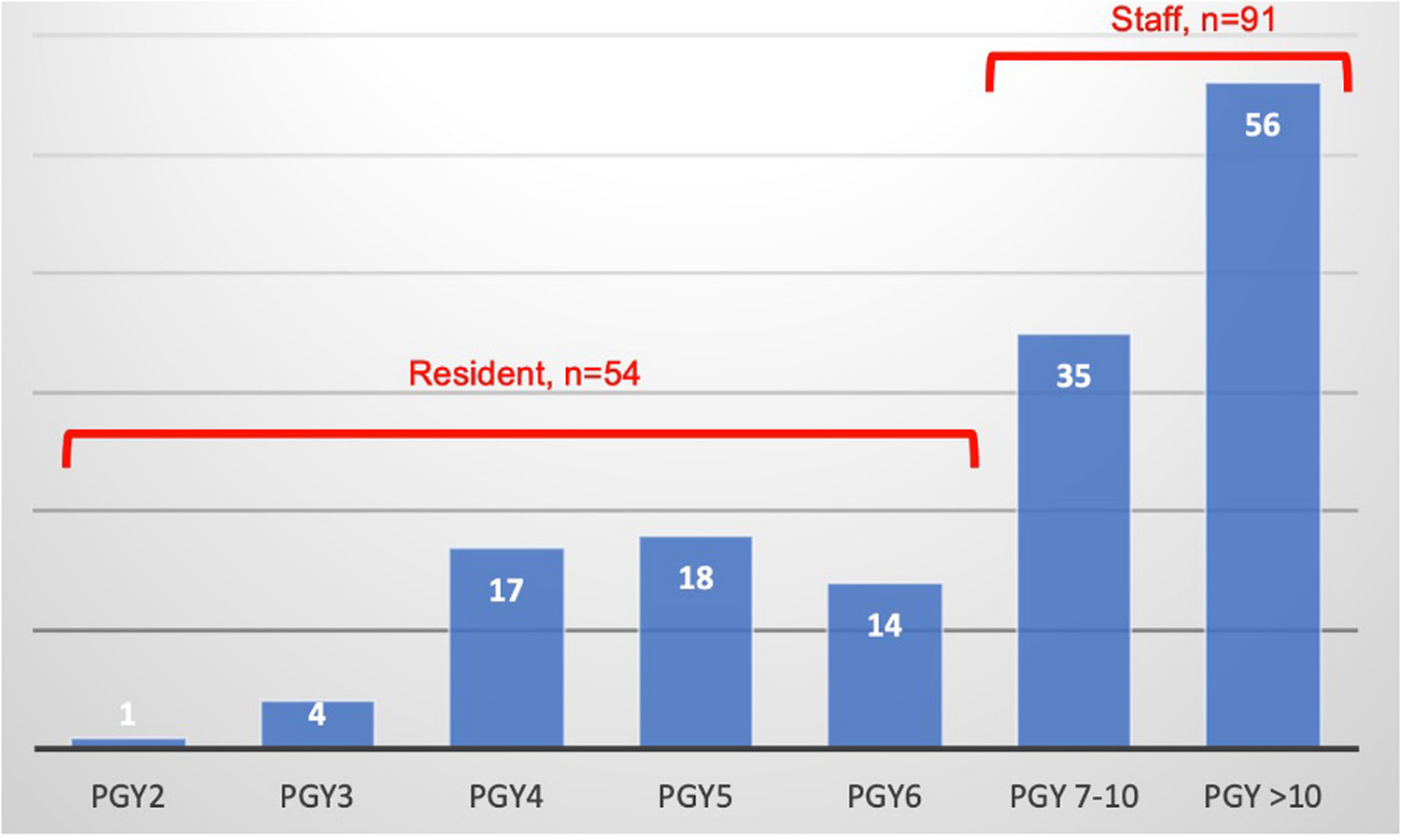
The number of cases performed by each postgraduate year.

This study was done retrospectively and was conducted independently at the Asahikawa Medical University. We included 145 patients undergoing isolated SAVR, at our institute, from January 2015 and April 2024. Patients were excluded if they underwent AVR combined with other concomitant procedures (i.e., coronary artery bypass grafting, other valvular procedures) or if they had had previous heart surgery. Patients having concomitant valve surgery or other concurrent cardiac surgical procedures were also excluded from this study. In the current study, the preoperative characteristics, early outcomes, and mid-term survival rates were compared between a group of patients who had undergone surgery by surgeons in their fifth year of training or later, i.e., older than 7th postgraduate year in the Japanese residency program (Staff, *n* = 91) and a group of patients who had undergone surgery by surgeons in their fourth year of training or younger, i.e., between 2nd and 6th postgraduate year in the Japanese residency program (Resident, *n* = 54). Each patient was discussed at the preoperative conference. Those with relatively minimal calcification of the ascending aorta, without a small annulus, and a low risk according to the Japan SCORE are assigned to the residents. Japan SCORE is a surgical risk score model specifically developed in Japan for cardiovascular surgery, based on the Japan Congenital Cardiovascular Surgery Database (JCVSD). It's designed to predict mortality, 30-day mortality, and the risk of major complications in patients undergoing cardiovascular surgery. In the Resident group, the staff surgeon was the first assistant, while the primary surgeon performed the entire operation from start to finish. In the Staff group, the assistant could perform some parts of the operation except those during cardiac arrest, depending on the case. The criteria for a young surgeon to qualify for performing operations are evaluated by the chief surgeon at our institute based on their proficiency in sternotomy, CPB establishment, and dry or wet lab training. Figure 1 illustrates the number of cases performed by each postgraduate year.

### Statistical analysis

Results are expressed as means ± standard deviation. Continuous variables exhibiting a normal distribution were tested using the *t*-test and continuous variables exhibiting a non-normal distribution were tested using the Mann–Whitney *U*-test. For categorical variables, Fisher's test was used. The log-rank test was used to determine the survival rate. Statistical significance was set at *p* < 0.05. Statistical analyses were performed using SPSS 22.0 software (SPSS Inc., Chicago, IL, USA), JMP software (JMP, Cary, North Carolina, USA) and Stata software (StataCorp, College Station, Texas, USA).

### Ethical standards

This retrospective study was approved by the institutional review board (No. 22161), which waived the need for written patient consent because of the retrospective nature of this study. Furthermore, the refusal right was warranted for all patients, as documented on our homepage.

## Results

The patients’ preoperative characteristics are shown in Table 1. The mean age of the patients was 74.5 ± 4.5 in the Staff group and 72 ± 5.0 in the Resident group (*p* = 0.103). The male ratio was 45.1% in the Staff group and 77.8% in the Resident group (*p* <0.01). Regarding comorbidity, there were no significant differences. As for the urgency, there were no differences between two groups. (urgent: 1 case (1.1%) in the Staff group vs. 1 case (1.9%) in the Resident group; *p* = 0.758 and emergent: 4 cases (4.4%) in the Staff group vs. 0 case (0%) in the Resident group; *p* = 0.153). Two urgent cases were both vital unstable aortic valve stenosis (AS) patients. As to emergent cases, three cases were infectious endocarditis (IE) patients, and one case was unstable aortic valve regurgitation (AR) patient. Japan SCORE showed significantly higher in Staff group (mortality: 7.53 ± 6.03% in the Staff group vs. 2.71 ± 2.59% in the Resident group; *p* = 0.029, mortality & morbidity: 18.7 ± 5.16% in the Staff group vs. 10.9 ± 5.16% in the Resident group; *p* = 0.005).

**Table 1:** Patients’ characteristics and preoperative data.

Variable	Staff	Resident	*P*-value
	*n* = 91	*n* = 54	
Age, mean ± SD years	74.5 ± 4.5	72.0 ± 5.0	0.103
Male gender, *n* (%)	41 (45.1)	42 (77.8)	<0.01
BMI, kg/m, mean ± SD	23.2 ± 2.0	22.4 ± 2.1	0.334
Comorbidity, *n* (%)			
Chronic renal disease (Cr ≧ 1.5)	16 (17.6)	7 (13.0)	0.486
COPD	5 (5.5)	4 (7.4)	0.749
Diabetes mellitus	21 (23.1)	9 (16.7)	0.356
CVA	3 (3.3)	0 (0)	0.278
DLP	25 (27.5)	13 (24.1)	0.658
Coronary artery disease	9 (9.9)	1 (1.9)	0.088
HTN	40 (44.0)	31 (57.4)	0.146
Af	5 (5.5)	3 (5.6)	0.983
Peripheral vascular disease	5 (5.5)	3 (5.6)	0.983
Urgency, *n* (%)			
Elective	86 (94.5)	53 (98.1)	0.412
Urgent	1 (1.1)	1 (1.9)	0.758
Emergent	4 (4.4)	0 (0)	0.153
Echocardiography			
Ejection fraction, ±SD %	60 ± 5.0	58 ± 7.0	0.794
Japan SCORE, *n* (%)			
Mortality, mean ± SD years	7.53 ± 6.03	2.71 ± 2.59	0.029
Mortality & morbidity, mean ± SD years	18.7 ± 5.16	10.9 ± 5.16	0.005

BMI, body mass index; COPD, chronic obstructive pulmonary disease; CVA, Cerebral vascular accident; DLP, dyslipidemia HTN, hypertension; Af, atrial fibrillation.

**Figure 2:**
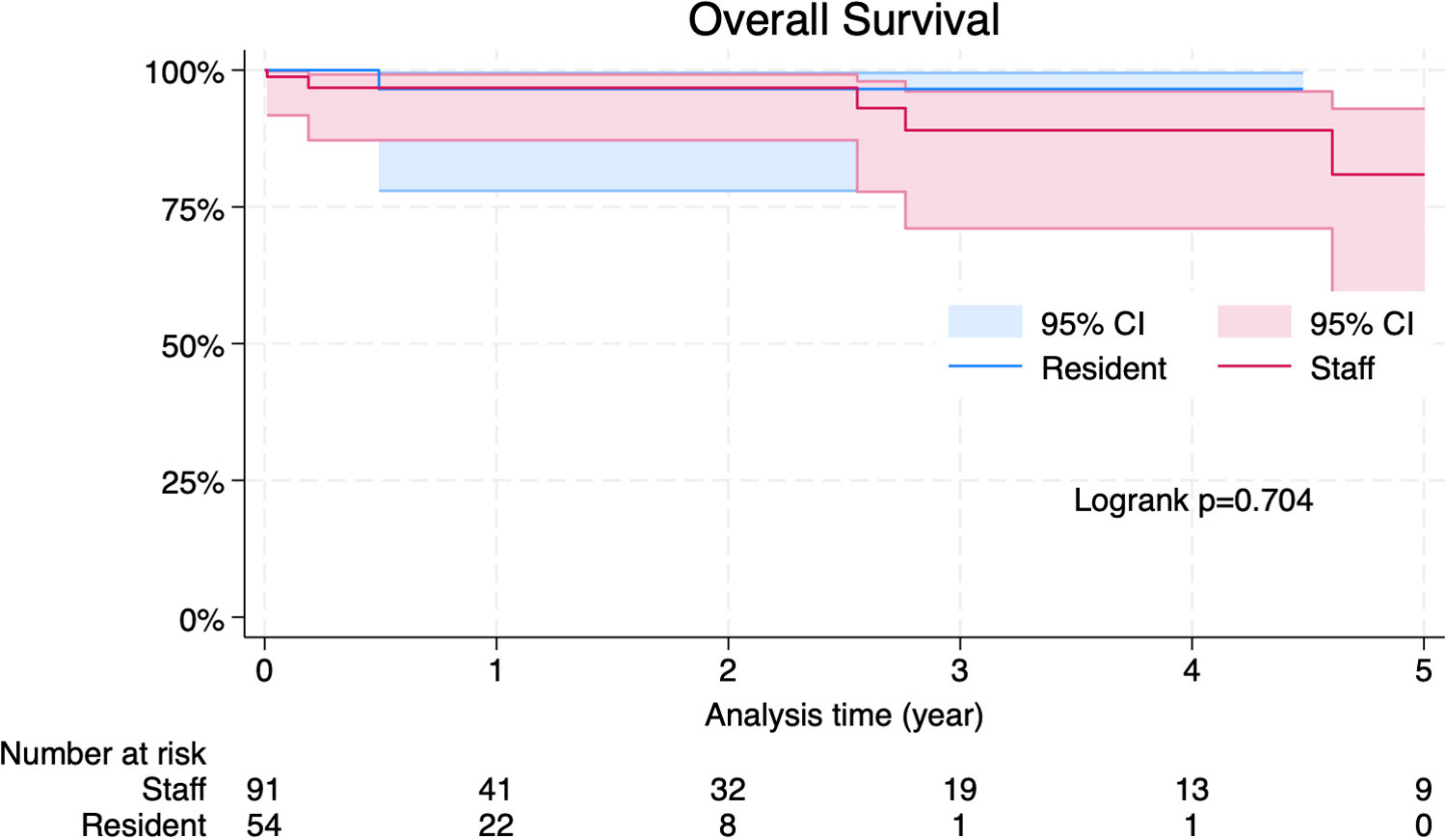
The survival curves.

Intraoperative characteristics are summarized in Table 2. As for the indications for SAVR, there were no differences between two groups. (AS: 74.7% in the Staff group vs. AS: 77.8% in the Resident group; *p* = 0.841 and AR: 22.0% in the Staff group vs. AR: 29.6% in the Resident group; *p* = 0.325). There were no differences regarding the ratio of bioprosthetic valves (89.0% in the Staff group vs. 88.9% in the Resident group; *p* = 1.000) and the size of the implanted prostheses (22.1 ± 3.0 in the Staff group vs. 22.2 ± 1.9 in the Resident group; *p* = 0.733). No differences were found in the operative time (238 ± 62.2 min in the Staff group vs. 231.6 ± 57.1 min in the Resident group; *p* = 0.491), cardiopulmonary bypass (CPB) time (121 ± 29.9 min in the Staff group vs. 125 ± 31.7 min in the Resident group; *p* = 0.457) and aortic cross-clamping (AXC) time (86 ± 23.4 min in the Staff group vs. 93 ± 23.8 min in the Resident group; *p* = 0.093). Postoperative characteristics are shown in Table 3. A 30 days mortality shows no significant difference (2 cases in the Staff group vs. 0 case in the Resident group; *p* = 0.519). No differences were found in intensive care unit (ICU) stay (3.0 ± 1.0 days in the Staff group vs. 2.5 ± 0.5 days in the Resident group; *p* = 0.100), however hospital stay was longer in the Staff group than in the Resident group (15.5 ± 3.5 in the Staff group vs. 13 ± 2.0 in the Resident group; *p* = 0.015). There were no differences in other postoperative complications. During the follow-up period, eight patients in the Staff group and one patient in the Resident group died. The survival curves are shown in Figure 2. There was no significant difference in late survival between the groups (*p* = 0.704).

**Table 2:** Operative data.

Variable	Staff	Resident	*P*-value
	*n* = 91	*n* = 54	
AS	68 (74.7)	42 (77.8)	0.841
AR	20 (22.0)	16 (29.6)	0.325
CPB time, mean ± SD min	121 ± 29.9	125 ± 31.7	0.457
AXC time, mean ± SD min	86 ± 23.4	93 ± 23.8	0.093
Operation time, mean ± SD min	238 ± 62.2	231.6 ± 57.1	0.491
Transfusion, *n* (%)	70 (76.9)	46 (85.2)	0.285
Bioprosthetic valves, *n* (%)	81 (89.0)	48 (88.9)	1.000
Aortic valve size	22.1 ± 3.0	22.2 ± 1.9	0.733

AS, aortic valve stenosis; AR, aortic valve regurgitation; CPB, cardiopulmonary bypass; AXC, aortic cross-clamping.

**Table 3:** Postoperative characteristics.

Variable	Staff	Resident	*P*-value
	*n* = 91	*n* = 54	
30 days mortality, *n* (%)	2 (2.2)	0 (0)	0.519
Stroke	2 (2.2)	1 (1.9)	1.000
Newly required dialysis	0 (0)	0 (0)	N/A
Prolonged ventilation	1 (1.1)	0 (0)	1.000
Deep sternal wound infection	0 (0)	0 (0)	N/A
Reoperation for bleeding	4 (4.4)	2 (3.7)	1.000
Permanent Pacemaker	3 (3.3)	0 (0)	0.294
POAF	20 (22.0)	17 (31.5)	0.239
ICU stay, mean ± SD days	3.0 ± 1.0	2.5 ± 0.5	0.100
Hospital stay, ±SD days	15.5 ± 3.5	13.0 ± 2.0	0.015

POAF, post operative atrial fibrillation; ICU, intensive care unit.

## Discussion

Several previous studies have demonstrated that allowing trainee surgeons to perform cardiac surgery under good supervision is not associated with worse early or late outcomes (4–12). In this study, we demonstrated that short-term clinical outcomes and mid-term survival after isolated SAVR are not negatively affected even by very early-stage trainees if patients were selected at the preoperative conference properly. A comparison of preoperative characteristics between cases performed by Staff and Resident surgeons demonstrated no differences except for Japan SCORE. And there were no differences in the incidence of other comorbidities. Although individual patient characteristics did not demonstrate significant differences, the Japan SCORE revealed notable disparities in risk profiles, potentially reflecting subtle yet cumulative variations in patient factors. It should be noted, however, that the Japan SCORE is heavily influenced by variables such as hemodynamic stability and heart failure severity—parameters that were not incorporated in our analysis due to data limitations. Additionally, experienced surgeons likely achieved favorable outcomes despite operating on patients with higher predicted risks. However, it is noteworthy that the resident group also achieved outcomes that exceeded predicted values, suggesting that residents can perform effectively when appropriately trained and supervised. This finding underscores the effectiveness of our training program and highlights the potential for residents to deliver high-quality care even in complex cases.

In general, trainees sometimes experience extended cardiopulmonary bypass and aortic cross-clamp times. This is understandable, as a decrease in intraoperative efficiency is an inevitable aspect of resident training. However, according to a study demonstrated by Haan et al. (10), the increased perfusion and bypass time associated with resident training does not compromise early outcomes. In fact, trainee surgeons achieved excellent early outcomes in our study. Contrary to previous reports, no differences were found in the operative time, CPB time and AXC time. Additionally, our data showed no difference of early-term and mid-term mortality between groups. Elizabeth D Krebs et al. (11) compared the outcomes of resident regarding the various aspects of cardiac surgery including AVR, showing that observed-to-expected ratios for mortality and combined morbidity-mortality were 0.66 and 0.72, respectively, and each individual resident exhibited better than predicted outcomes. Our data shows similar findings and suggests that training status does not increase the risk of early mortality after AVR under proper supervision and with appropriate case selection. A comparison of postoperative complications also demonstrated similar outcomes between the two groups. Furthermore, training status was not associated with an increased risk of stroke, new renal failure, prolonged ventilation, deep sternal wound infection, reoperation for bleeding, permanent pacemaker and post operative atrial fibrillation (POAF). The Staff group surgeons were Board Certified Surgeon who had graduated from a medical school for at least seven years. In the Resident group, no surgeons were Board Certified, and they had graduated from a medical school within two to six years. Although ensuring patient safety is the foremost priority in surgical education, it is also crucial to employ effective educational methods to cultivate young surgeons and enhance their interest and motivation in surgery. In this study, no negative impact on surgical outcomes was observed between the groups of staff and resident surgeons in the context of SAVR. This suggests that safety can be maintained under the supervision of experienced surgeons. It may be important to establish quantifiable criteria for preoperative case selection as a next step in future research.

### Limitations

There are several limitations to the present study. First, this was nonrandomized and retrospective study of a single center's experience with a relatively small sample size. Therefore, there may persist a certain degree of selection bias and potential confounding which could have influenced our findings.

Additionally, there was a certain degree of variability in the number of years and experience among the staff group surgeons. Regarding the shorter follow-up period observed in the trainee group, this may be attributable to the limited number of younger doctors without board certification until recently.

Finally, in the case of the staff surgeon group, the trainee performed some parts of operation except during cardiac arrest in some cases, and it cannot be denied that this may have affected the operation time and post operation outcomes.

## Conclusion

In conclusion, AVR can be safely performed by very early-stage trainees under proper supervision. Trainee status is not associated with an increased risk of short-term mortality and is associated with a similar incidence of most post-operative complications.

## Data Availability

The raw data supporting the conclusions of this article will be made available by the authors, without undue reservation.
